# Diversity of *Cryptosporidium* species occurring in sheep and goat breeds reared in Poland

**DOI:** 10.1007/s00436-016-5360-3

**Published:** 2017-01-05

**Authors:** Agnieszka Kaupke, Mirosław M. Michalski, Artur Rzeżutka

**Affiliations:** 1 Department of Food and Environmental Virology, National Veterinary Research Institute, al. Partyzantów 57, 24-100 Puławy, Poland; 2 Department of Parasitology and Invasive Diseases, Faculty of Veterinary Medicine, University of Warmia and Mazury, Oczapowskiego 13, 10-719 Olsztyn, Poland

**Keywords:** *Cryptosporidium*, Prevalence, Sheep, Goat, Breed

## Abstract

The aim of this study was molecular identification of *Cryptosporidium* species and assessment of their prevalence in different breeds of sheep and goat reared in Poland. In addition, the relationship between animal age, breed type, and the frequency of *Cryptosporidium* infections was determined. Fecal samples from 234 lambs and 105 goat kids aged up to 9 weeks, representing 24 breeds and their cross-breeds were collected from 71 small ruminant farms across Poland. The identification of *Cryptosporidium* species was performed at the 18 SSU ribosomal RNA (rRNA) and COWP loci followed by subtyping of *C. parvum* and *C. hominis* strains at GP60 gene locus. The presence of *Cryptosporidium* DNA at the 18 SSU rRNA locus was detected in 45/234 (19.2%) lamb feces samples and in 39/105 (37.1%) taken from goats. The following *Cryptosporidium* species: *C. xiaoi*, *C. bovis*, *C. ubiquitum*, *C. parvum,* and *C. hominis* were detected in small ruminants. Infections caused by *C. xiaoi* were predominant without favoring any tested animal species. Subsequent GP60 subtyping revealed the presence of *C. parvum* IIaA17G1R1 subtype in sheep and IIdA23G1 subtype in goats. IIdA23G1 subtype was detected in a goat host for the first time. There were no significant differences found in frequency of infections between the age groups (<3 and 3–9 weeks) of lambs (*P* = 0.14, α > 0.05) or goat kids (*P* = 0.06, α > 0.05). In addition, there was no correlation observed between the frequency in occurrence of particular parasite species and breed type in relation to native sheep breeds (*F* = 0.11; *P* = 0.990 > 0.05). In the case of goats, more breed-related differences in parasite occurrence were found. The results of this study improve our knowledge on the breed-related occurrence of *Cryptosporidium* infections in the population of small ruminants reared in Poland.

## Introduction

Cryptosporidiosis has been described in a variety of farm animals including sheep and goats. Some data exists on the *Cryptosporidium* species/genotypes infecting small ruminants reared all over the world (Karanis et al. 2007; Mueller-Doblies et al. 2008; Pritchard et al. 2008; Quílez et al. 2008; Paoletti et al. 2009; Robertson et al. 2010; Díaz et al. 2010; Imre et al. 2013; Connelly et al. 2013; Rieux et al. 2013; Tzanidakis et al. 2014; Yang et al. 2014; Díaz et al. 2015). It reveals the prevalence of *C. ubiquitum*, *C. xiaoi*, and *C. parvum* to be the highest in these animal hosts. However, depending on the geographical region, other *Cryptosporidium* species such as *C. andersoni* (sheep and goats), *C. hominis* (goats), *C. bovis* (sheep), and occasionally *C. scrofarum* and *C. suis* may also be isolated (Koinari et al. 2014; Yang et al. 2014). Small ruminants are also known as a reservoir of *C. hominis* (Ryan et al. 2005; Giles et al. 2009; Connelly et al. 2013; Kang’ethe et al. 2012; Koinari et al. 2014) and zoonotic *C. parvum* for humans (Geurden et al. 2008; Quílez et al. 2008; Paoletti et al. 2009; Robertson 2009; Imre et al. 2013; Cacciò et al. 2013; Lange et al. 2014; Díaz et al. 2015; Taylan-Ozkan et al. 2016). Although infections are frequently reported, their impact on animal health seems to be of less importance than in cattle. Usually, *Cryptosporidium* infections in small ruminants are asymptomatic and are only rarely accompanied by gastrointestinal disorders. The symptomatic course of *Cryptosporidium* infection is characterized by neonatal diarrheal diseases which are associated with *C. parvum* and sporadically with *C. ubiquitum* or *C. xiaoi* infections (Imre et al. 2013).

The significant economic losses in sheep and goat breeding attributed to parasitic infections are due to invasion of coccidia (Vasilkova et al. 2004; Balicka-Ramisz et al. 2012), sheep tapeworm (*Moniezia* spp.), and nematodes from the *Trichostrongylidae* family (Malczewski 1970; Buddle et al. 1988). However, the main causes of morbidity and mortality among small ruminants are bacterial or viral infections complicated by parasites including *Cryptosporidium* (Mason et al. 1981; Ozmen et al. 2006). Although several studies aimed at detecting and identifying *Cryptosporidium* in farm animals have been conducted, our knowledge of parasite occurrence and worldwide distribution in animals is still not complete (Plutzer and Karanis 2009; Xiao 2010). For example, studies concerning cryptosporidiosis in small ruminants were mostly focused on parasite detection using microscopic methods without subsequent identification of parasite species. Therefore, little is known about the epidemiology of *Cryptosporidium* infections caused by particular parasite species and their occurrence in the population of small ruminants in Eastern European countries. Data is also lacking on transmission dynamics within animal population, age, or breed-related parasite occurrence (Díaz et al. 2015).

Currently, sheep farming in Poland is not pursued on the same scale as in the 1980s, when the sheep population reached its maximum size of nearly 5 million animals. At the same time, goat raising was also thriving; however, it recorded a 39% fall in 2002 and the trend continues up to the present. The number of small ruminants in Poland amounts to 268,000 sheep and 117,500 goats. Mostly, goat husbandry is in small flocks of up to 4 animals, or 20 in the case of sheep (CSO 2010). The sheep and goats raised in Poland represent 30 and 10 breed types, respectively. The most common sheep breeds are Polish Heath (WRZOS), Pomeranian Coarsewool (POM), and Wielkopolska sheep (WLKP) because of their meat and wool quality. Three breeds constitute the largest goat population in Poland, i.e., Polish White Improved (BIALA USZL), Polish Color Improved (BARWNA USZL), and Saanen (SAAN).

The aim of this study was the identification and assessment of the prevalence of *Cryptosporidium* species in different breeds of sheep and goat in husbandry in Poland using molecular methods. In addition, the relationship between animal age, breed type, and the frequency of *Cryptosporidium* infections was determined.

## Materials and methods

### Source of samples and study design

During the three-year period 2011–2016, 234 lamb feces samples and 105 goat kids feces were collected from animals from birth up to the age of 9 weeks. The animals were housed in 61 sheep and 11 goat farms located in several administrative provinces of Poland where breeding of these animals is a tradition. The farms visited were operated according to a traditional grazing-based husbandry system. Farms and animals were randomly chosen for sampling. On the day of sampling, all animals were in good health without any visible symptoms of disease. All animals were periodically dewormed. The number of sampled animals per farm ranged from 3 to 4 in the case of lambs and from 5 to 10 for goat kids. The sampled lambs belonged to 16 breeds and their cross-breeds: Wielkopolska sheep (WLKP), Polish Mountain sheep (POG), Uhruska sheep (UHR), Kamieniecka sheep (KAM), Pomeranian Coarsewool (POM), Polish Heath (WRZOS), Blackhead Persian (CZGL), Polish Merino (MP), Olkuska Sheep (OLK), Suffolk (SUF), Bergschaf (BERG), Polish Lowland (PON), Whitehead Sheep (BOM), Żelazna Sheep (ŻEL), Podhale Sheep (CKP), and meat cross-breeds (MM).

The goat kids represented six breeds including meat cross-breeds (MK): Saanen (SAAN), Alpine (ALP), Polish White Improved (BIALA USZL), Polish Color Improved (BARWNA USZL), and Anglo-Nubian (ANGL-NUB). Upon collection, feces were placed into plastic containers, labeled, and sent to the laboratory. Before analysis, they were stored for a maximum of 1 week at 4 °C, or at −20 °C, if processing was delayed more than 1 week.

### Molecular detection and species identification

Parasite genomic DNA was extracted from 0.1 g of feces using a previously described method (Rzeżutka and Kaupke 2013). The correct performance of the method was monitored by a positive extraction control (feces of sheep contaminated with *C. parvum* oocysts (Iowa strain, Waterborne™, Inc., New Orleans, LA, USA)) and a negative control (water instead of the analyzed template). These controls were included for each set of analyzed samples and simultaneously processed. The nucleic acids were subjected to further purification with the use of a GeneMATRIX PCR/DNA Clean-Up Purification Kit, (EURx, Ltd., Gdańsk, Poland) as recommended by the manufacturer. The extracts containing parasite DNA were stored at −20 °C until use.

The identification of *Cryptosporidium* species was performed at the 18 SSU ribosomal RNA (rRNA) and COWP loci and was followed by subtyping at the GP60 gene locus (Homan et al. 1999; Xiao et al. 1999; Glaberman et al. 2002; Sulaiman et al. 2005). Subsequently, a restriction fragment length polymorphism (RFLP) analysis was performed for all positive 18 SSU rRNA and COWP PCR products. The analyses of 18 SSU rRNA amplicons were conducted using *Nde*I for initial identification and differentiation of *C. parvum* and *C. hominis* infections from other species infecting small ruminants and *Xba*I for differentiation of *C. ubiquitum* (lack of restriction site) from *C. bovis* and *C. xiaoi* (Xiao et al. 1999, 2006; Zintl et al. 2007). In addition, the COWP amplicons were subjected to *Taq*I digestion for definitive confirmation of *C. parvum* or *C. hominis* presence (Homan et al. 1999). In addition to controls included during DNA isolation, the appropriate positive and negative controls were also included during PCR analyses.

Visualization of PCR amplicons and their restriction patterns was conducted in either 1.7 or 2.5% agarose gel stained with ethidium bromide. A definitive identification of *Cryptosporidium* species revealing the same “group”-specific restriction pattern (*C. ubiquitum* and *C. bovis)* was obtained on the basis of sequencing results. The 18 SSU rRNA amplicons were purified and sequenced, and their consensus sequences were compared with those available in the GenBank database as previously described (Rzeżutka and Kaupke 2013).

### Statistical analyses

The relationship between the age of the lambs and goat kids and frequency of *Cryptosporidium* occurrence, and the dominance of infections caused by *C. xiaoi*, *C. bovis,* and *C. ubiquitum* in the tested sheep population were analyzed using one-way analysis of variance (ANOVA). A chi-square (*χ* ^2^) test was also used for showing the relationship between the occurrence of infection and animal age. A two-way analysis of variance without interactions with Tukey confidence intervals (CI) allowed demonstration of the differences in frequency of occurrence of particular parasite species in native Polish sheep breeds (POG, WLKP, KAM, WRZOS, MP, and UHR). Finally, the influence of sheep and goat breed on frequency of infections was investigated. All calculations were performed with a Statgraphics Centurion v. XV (Statpoint Technologies, Warrenton, USA).

## Results

### Molecular identification of *Cryptosporidium* spp.

The presence of *Cryptosporidium* DNA at the 18 SSU rRNA locus was detected in 45/234 (19.2%) and in 39/105 (37.1%) of lamb and goat feces, respectively. Successful amplification of the COWP gene fragment was obtained only for 2 samples, although previous 18 SSU rRNA PCR-RFLP analysis did not indicate the possible presence of *C. parvum* or *C. hominis* DNA. Subsequent GP60 subtyping revealed the presence of IIaA17G1R1 *C. parvum* subtype in sheep and IIdA23G1 subtype in goat. Because of primer mismatches within the GP60 gene fragment, subtyping was unsuccessful for one *C. parvum* and one *C. hominis* strain. Despite multiple attempts, two *C. parvum* and *C. hominis* sheep isolates could not be successfully identified at the GP 60 locus, due to poor homology between forward and reverse primer sequences.

The RFLP and subsequent sequence analysis of all 18 SSU rRNA and COWP amplicons allowed identification in sheep of the following *Cryptosporidium* species: *C. xiaoi* (*n* = 33), *C. bovis* (*n* = 9), *C. ubiquitum* (*n* = 3), *C. parvum* (*n* = 2), *C. hominis* (*n* = 1), and *Cryptosporidium* spp. (*n* = 1). Goats were infected by *C. xiaoi* (*n* = 29) and *C. parvum* (*n* = 1). Animals were mostly infected by a single parasite species, except for three lambs in which mixed infections of two (*C. xiaoi*/*C. parvum* or *C. xiaoi*/*Cryptosporidium* spp.) or three (*C. xiaoi*/*C. parvum*/*C. hominis*) parasites were noticed. The representative 18 SSU rRNA nucleotide sequences of each species detected in sheep and goats from different age groups and breed types were deposited in GenBank under the accession numbers listed in Table 1.

**Table 1:** Prevalence of *Cryptosporidium* species in association to breed and age of tested animals

Animal species	Breed	Number of samples (positive/tested)	*Cryptosporidium* infection in age groups			
			1 day–3 weeks	GeneBank accession no.	>3–9 weeks	GeneBank accession no.
Sheep	MP	10/39	*C. xiaoi* (*n* = 2^a,b^)	KY055383	*C. ubiquitum* (*n* = 1)	KY055388
			*Cryptosporidium* spp. (*n* = 1^a^)		*C. xiaoi* (*n* = 7)	KY055389, KY055396
			*C. parvum* (*n* = 1^b^)	KY055411		
			*C. hominis* (*n* = 1^b^)	KY055410		
	POG	4/31	*C. xiaoi* (*n* = 2)	KY055387	*C. xiaoi* (*n* = 2)	KY055394
	WLKP	12/24	*C. bovis* (*n* = 6)	KY055380, KY055381	–	
			*C. xiaoi* (*n* = 1^c^)	KY055382		
			*C. parvum* (*n* = 1^c^)	KY055409		
	UHR	4/24	*C. xiaoi* (*n* = 1)	KY055376	*C. bovis* (*n* = 1)	KY055378
					C. xiaoi (*n* = 2)	KY055377
	WRZOS	7/24	*C. ubiquitum* (*n* = 1)	KY055397	*C. xiaoi* (*n* = 6)	KY055385, KY055386
	KAM	2/23	*C. xiaoi* (*n* = 2)	KY055384, KY055390	–	
	OLK	0/16	–		–	
	MM	3/14	*C. ubiquitum* (*n* = 1)	KY055391	–	
			*C. xiaoi* (*n* = 2)	KY055392		
	CZGL	2/11	*C. xiaoi* (*n* = 1)	KY055393	*C. bovis* (*n* = 1)	KY055379
	PON	1/8	–		*C. xiaoi* (*n* = 1)	KY055395
	POM	0/4	–		-	
	ZEL	0/4	–		-	
	CKP	0/3	–		-	
	SUF	0/3	–		-	
	BOM	0/2	–		-	
	BERG	0/1	–		-	
Goat	SA	12/25	*C. xiaoi* (*n* = 3)	KY055399	*C. xiaoi* (*n* = 9)	KY055401
	AL	11/18	–		*C. xiaoi* (*n* = 11)	KY055398, KY055400, KY055404
	BIALA USZL	2/4	–		*C. xiaoi* (*n* = 2)	KY055402
	BARWNA USZL	3/20	*C. xiaoi* (*n* = 2)	KY055406	*C. xiaoi* (*n* = 1)	KY055405
	AN	7/8	*C. xiaoi* (*n* = 2)	KY055403	*C. xiaoi* (*n* = 5)	KY055407
	KB	4/30	*C. parvum* (*n* = 1)	KY055412	–	
			*C. xiaoi* (*n* = 3)	KY055408	–	

^a^Mixed infection *C. xiaoi* / *Cryptosporidium* spp. (not identified species)

^b^Mixed infection *C. xiaoi* / *C*. *parvum* / *C. hominis*

^c^Mixed infection *C. xiaoi* / *C*. *parvum*

### Distribution of *Cryptosporidium* spp. related to animal age and breed

The infected lambs and goat kids were kept on 25 (41%) and 7 (63.6%) farms out of the 61 and 11 respectively investigated. *C. xiaoi* was found on 22 (36%), *C. bovis* on 4 (6.5%), *C. ubiquitum* on 3 (4.9%), *C. parvum* on 2 (3.2%), and *C. hominis* on 1 (1.6%) sheep farm. On seven (63.6%) out of 11 goat farms, *C. xiaoi* was detected, whereas *C. parvum* was only found on 1 (9%) farm.

**Fig. 1:**
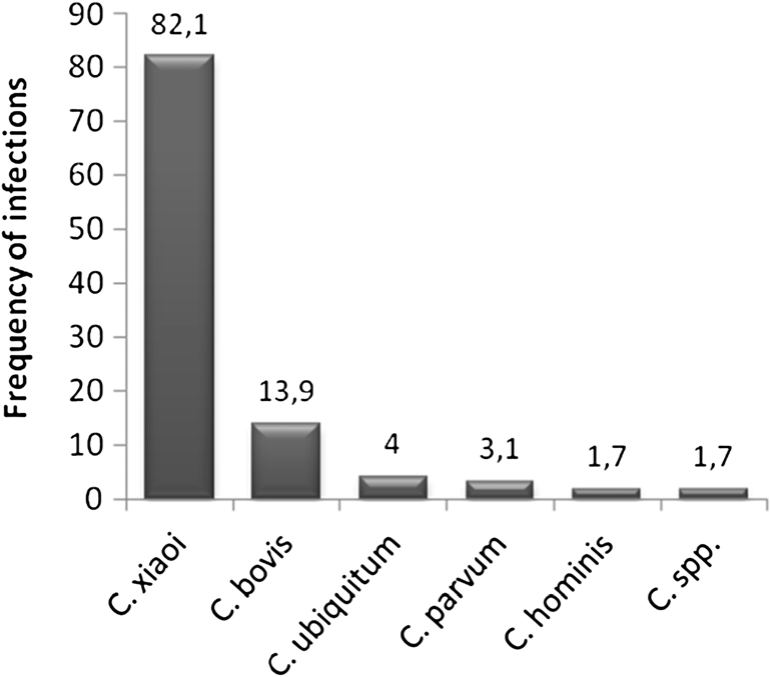
The frequency of infections caused by particular *Cryptosporidium* species in the tested sheep population

**Fig. 2:**
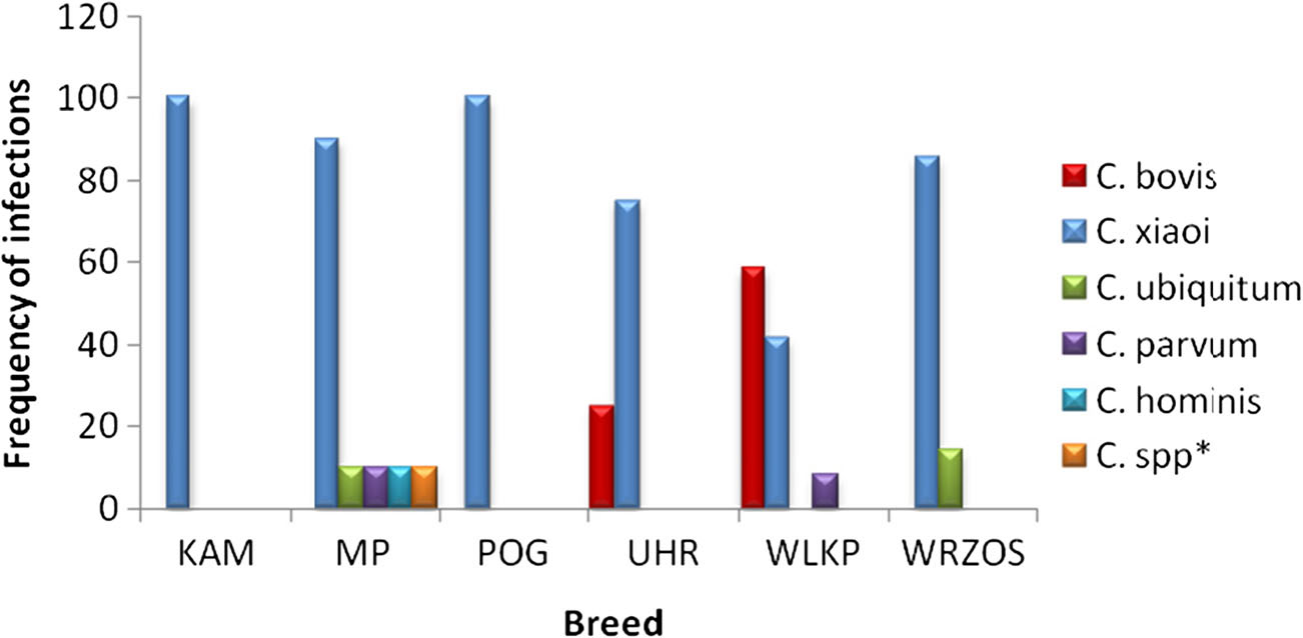
The frequency of infections caused by particular species of the parasite in relation to the tested sheep breeds

The analysis of variance showed that the frequency of infections did not differ significantly (*P* = 0.14, α > 0.05) between the tested age groups of lambs (<3 and 3–9 weeks). The prevalence of the parasite among the youngest animals was 15.57% but was 23.21% in animals older than 3 weeks. However, the difference between these values was not statistically significant (NIR_0.05_ = 10.16%). Also, there was no significant correlation observed between the presence of infection and animal age (*χ* ^2^ = 2.195, *P* = 0.139 > 0.05). Neither were there any significant differences found in the frequency of infections in goats between the age groups (*P* = 0.06, α > 0.05), with the infection prevalence within each group at 26.2 and 44.4%. Infections caused by *C. xiaoi* were predominant without favoring any tested animal species. They were detected in 33 (14.1%) of lambs and 29 (27.6%) goat kids. *C. xiaoi* prevailed (82.1%) in sheep, and the frequency of its occurrence was significantly higher (*F* = 26.99, *P* = 0.000 < 0.05) than those of the infections caused by other parasite species such as *C. bovis*, *C. ubiquitum*, *C. parvum*, or *C. hominis* (Fig. 1). *C. xiaoi* was not only prevalent at herd level but also was the most often occurring parasite at farm level despite their dispersed locations. The least frequently detected species were *C. parvum* and *C. hominis* which were responsible for less than 3.1 and 1.7% of infections, respectively. There was no correlation observed between the frequency in occurrence of particular parasite species and breed in relation to native sheep breeds (*F* = 0.11; *P* = 0.990 > 0.05). The frequency of *Cryptosporidium* occurrence in particular sheep breeds is presented in Fig. 2.

*Cryptosporidium* infections were most frequently observed in WLKP in comparison to KAM and POG (*F* = 3.23, *P* = 0.008 < 0.05). The frequencies of infections for other Polish breeds (UHR, MP, and WRZOS) did not differ significantly from each other. In the case of goats, more breed-related differences in parasite occurrence were found. *Cryptosporidium* was most frequently detected in ALP, ANGL-NUB, and SAAN than in animals of the BARWNA USZL and MK breeds (*F* = 6.57, *P* = 0.001 < 0.05).

## Discussion

Nowadays, molecular methods are widely used in diagnosis of *Cryptosporidium* infections in farm animals (Cacciò et al. 2013) including population and epidemiological studies (Geurden et al. 2008; Fiuza et al. 2011). So far, few studies aiming to identify and assess the parasite prevalence in small ruminants have been carried out in Europe and the majority of data come from France, Italy, England, and Romania (Misić et al. 2006; Mueller-Doblies et al. 2008; Quílez et al. 2008; Pritchard et al. 2008; Paoletti et al. 2009; Robertson et al. 2010; Díaz et al. 2010; Imre et al. 2013; Connelly et al. 2013; Cacciò et al. 2013; Rieux et al. 2013; Tzanidakis et al. 2014; Díaz et al. 2015). As seen in these studies, the worldwide prevalence of *Cryptosporidium* infections in sheep was in the range of 1.6 to 77.4% (Santín et al. 2007; Fiuza et al. 2011), while in goats, it was from 3.48 to 72.5% (Wang et al. 2014; Romero-Salas et al. 2016). This varied prevalence indicates a large geographical disparity in the frequency of infections that may be attributed to differences in animal age, breed, or the management and husbandry practices used (Mahfouz et al. 2014).

In this study, sheep and goats were carrying *Cryptosporidium* asymptomatically, with the extensiveness of invasion estimated at 19.2 and 37%, respectively. Infections in sheep in Poland were diagnosed more often than in sheep flocks farmed in Belgium, Greece, or Norway (Geurden et al. 2008; Robertson et al. 2010; Tzanidakis et al. 2014). However, the parasite prevalence was lower than that observed in sheep from Serbia, Turkey, England, and Australia (Ryan et al. 2005; Misić et al. 2006; Mueller-Doblies et al. 2008) and similar (17.45%) to the prevalence in Italian flocks (Paoletti et al. 2009). In contrast to its incidence in the Polish sheep population, *Cryptosporidium* occurrence in goats was higher than that observed in other European countries (Misić et al. 2006; Geurden et al. 2008; Tzanidakis et al. 2014). Although the frequency of infections did not differ statistically between the studied age groups of lambs, the number of infected animals increased with their age. For example, in lambs aged up to 3 weeks, the prevalence was 15.6% and in older lambs, it was 23.2%, while in goats, the prevalences were 26.2 and 44.4%, respectively. A similar increase in *Cryptosporidium* prevalence related to animal age was also observed by Misić et al. (2006) and Rieux et al. (2013).

Among identified *Cryptosporidium* spp.*, C. xiaoi*, *C. parvum*, *C. ubiquitum*, *C. andersoni*, *C. hominis*, *C. bovis*, *C. ryanae*, and *C. scrofarum* including the three genotypes rat, sheep I, and marsupial can infect small ruminants (Wang et al. 2010; Yang et al. 2014; Koinari et al. 2014; Li et al. 2016). Nevertheless, infections of sheep and goats are mainly caused by *C. xiaoi*, *C. parvum*, and *C. ubiquitum* (Geurden et al. 2008; Mueller-Doblies et al. 2008; Robertson et al. 2010; Yang et al. 2014; Mi et al. 2014; Paraud et al. 2014). These parasites were identified with different frequencies in sheep of different ages. Indeed, the most commonly *Cryptosporidium* detected in sheep in Europe was *C. ubiquitum* (Elwin and Chalmers 2008; Geurden et al. 2008; Robertson et al. 2010; Connelly et al. 2013; Tzanidakis et al. 2014), while in goats, the most evident numerically was *C. parvum* (Geurden et al. 2008; Tzanidakis et al. 2014), although their prevalence was not always the highest in the tested flock (Connelly et al. 2013; Tzanidakis et al. 2014). A similar distribution of *C. ubiquitum* has been found in sheep raised in North (Santín et al. 2007) and South America (Fiuza et al. 2011; Paz e Silva et al. 2014) and Asia (Wang et al. 2010; Shen et al. 2011; Ye et al. 2013), but not in Australia, where *C. xiaoi* was the predominant species (Sweeny et al. 2011; Yang et al. 2014).

*C. xiaoi* infections prevailed in small ruminants in Poland, regardless of animal age, breed type (except WLKP), or farm location. This species was mostly identified in healthy animals (Rieux et al. 2013; Mahfouz et al. 2014) although its occurrence can also be associated with diarrhea in lambs and goat kids (Imre et al. 2013; Díaz et al. 2010; Díaz et al. 2015). Similar prevalence of the parasite was found in sheep in France (Rieux et al. 2013) and in goat flocks in Greece (Tzanidakis et al. 2014). Unlike other authors’ observations, (Santín et al. 2007; Geurden et al. 2008; Wang et al. 2010), in this study, *C. ubiquitum* infections in lambs were characterized by low prevalence of 1.3%. In contrast to sheep, *C. ubiquitum* was not identified in goats, although it has been previously detected in both newborn kids and adult goats (Tzanidakis et al. 2014; Mi et al. 2014; Wang et al. 2014). As demonstrated in previous studies, *C. bovis* has occasionally been identified in lambs (4.8%) (Santín et al. 2007) and older animals up to the age of 2 years (1.1%) (Mueller-Doblies et al. 2008). A similar 5.5% prevalence of *C. bovis* in lambs was also detected in Poland. Within Polish cattle herds, it was the most widely occurring species in young calves (Rzeżutka and Kaupke 2013). The high similarity (99.5%) of sequences of the *C. bovis* strains derived from cattle and sheep raised in the same regions must signify the possibility of parasite exchange between ruminants and the importance of the environment in their transmission (data not shown).

Infections caused by *C. parvum* in sheep and goats can result in diarrhea (Quílez et al. 2008; Imre et al. 2013; Cacciò et al. 2013; Díaz et al. 2015) associated with a high occurrence of the parasite affecting up to 100% of individuals in the flock (Quílez et al. 2008). Nevertheless, when asymptomatic carriage of *C. parvum* has been reported, the extensiveness of invasion was low (0.73% in sheep) (Geurden et al. 2008) or moderate (20.3% in goats) (Mi et al. 2014). The differences observed in the course of infection could be attributed to different pathogenicity of *C. parvum* strains. In this study, *C. parvum* was detected in 0.85% in sheep and 0.95% in goats aged up to 3 weeks. The low age of asymptomatically infected animals is consistent with the observations of Ryan et al. (2005) and Santín et al. (2007). Surprisingly, in a previous study carried out in Poland, 10.1% prevalence of *C. parvum* in lambs older than 3 months was observed in sheep flocks in the Wielkopolska region (Majewska et al. 2000). Unlike our results, in that study, other parasite species commonly occurring in small ruminants were not identified, probably due to limitations of the methods used.

Cryptosporidiosis does not constitute a major health problem in sheep and goats. However, infected animals can be reservoirs of zoonotic *Cryptosporidium* species for humans (Lange et al. 2014). *C. parvum*, *C. hominis*, *C. ubiquitum,* and *C. andersoni* infections in ruminants are of particular importance, due to the zoonotic nature of these parasites (Hijjawi et al. 2010; Cieloszyk et al. 2012; Cacciò et al. 2013; Jiang et al. 2014). Among several *C. parvum* genetic families, strains belonging to the IIa and IId families cause disease in humans and animals (Xiao and Ryan 2004; Abe et al. 2006; Plutzer and Karanis 2009). In this study, the presence of the IIaA17G1R1 subtype in lambs and IIdA23G1 in goat kids was demonstrated. Apart from sheep, IIaA17G1R1 has previously been detected in cattle in Poland and other countries in Europe (Stantic-Pavlinic et al. 2003; Wielinga et al. 2008; Plutzer and Karanis 2007; Brook et al. 2009; Imre et al. 2013; Kaupke and Rzeżutka 2015). This strain was among two other subtypes (IIaA15G2R1 and IIaA16G1R1b) mostly infecting cattle in Poland (Kaupke and Rzeżutka 2015). A common subtype occurrence in both cattle and sheep indicates the possibility of parasite transmission between different species of ruminants kept in the same area. In addition, the importance of the IIaA17G1R1 *C. parvum* subtype in the epidemiology of human cryptosporidiosis has previously been shown (Soba and Logar 2008; Sharbatkhori et al. 2015). For the first time, the IIdA23G1 subtype was detected in a goat host. However, it has been reported in cattle herds in Sweden, Spain, and Poland (Silverlås et al. 2010; Quílez et al. 2011; Kaupke and Rzeżutka 2015). *C. hominis* infections in small ruminants are rare (Connelly et al. 2013; Koinari et al. 2014), and this observation has also been confirmed by our finding of *C. hominis* DNA in a single stool sample originating from a 3-week-old lamb. In sheep, there were also mixed infections detected caused by two (*C. xiaoi/C. parvum, C. ubiquituum/C. parvum, C. ubiquituum/C. bovis*) or three (*C. xiaoi/C. parvum/C. hominis*) *Cryptosporidium* species. In fact, mixed infections were only sporadically observed in sheep (Elwin and Chalmers 2008; Sweeny et al. 2011; Yang et al. 2014).

There is a lack of studies aiming to assess the relationship between the animal breed, occurrence of *Cryptosporidium* species, and the frequency of infections. In the current study, a more pervasive invasion was found in sheep of WLKP and MP breeds as well as in goats of AL breed. Likewise, the virulence of *Cryptosporidium* strains detected in sheep and goats appear to be of less importance due to the asymptomatic course of infections caused by them. The results may indicate the differences in sensitivity of individual sheep and goat breeds to *Cryptosporidium* infection. Contrary to this observation, Romero-Salas et al. (2016) suggested that the breed, age, or gender of animals have no significant impact on *Cryptosporidium* prevalence in small ruminants. Nevertheless, the sampled animals represented two goat (Mixed and Nubian) and three sheep breed types (Pelibuey, Dorper, and Kathadin) not raised in Europe, which to some extent could explain the observed differences. Nevertheless, different resistance of sheep to gastrointestinal nematode parasites in various breeding conditions and environments has been reported by Bouix et al. (1998). The goat breed was not indicated as a significant risk factor for *Cryptosporidium* infection in dairy goat farms in Western France (Delafosse et al. 2006).

Nowadays, sheep and goat breeding in Poland is not as popular as it was in the last century. The size of flocks is small, often comprising two or three animals (CSO 2010). Although the studies were conducted on randomly selected animals originating from different farms and locations, the number of tested animals did not reflect the size of the small ruminant population in the country. This could be taken as a major limitation in interpretation of the results on the occurrence of *Cryptosporidium* infections in sheep and goat herds in Poland. The results on the occurrence of *Cryptosporidium* species in the investigated sheep and goat breeds should also be interpreted with caution because the higher prevalence of a particular parasite species in the flock does not necessarily indicate the greater sensitivity of the animal breed to *Cryptosporidium* infection. It could be a result of an endemic occurrence of the parasite in this area. Certainly, if a higher number of animals representing particular breed type were tested, then data regarding the host-parasite interactions would be more evident.

## Conclusions

*Cryptosporidium* infections are widespread in lambs in spite of the age of animals, breed type, and farm location. The occurrence of *C. parvum* and *C. hominis* in small ruminants highlights the importance of these animal species in parasite circulation between animal and human hosts. However, the epidemiology of infections and the occurrence of other zoonotic species in small ruminants, with their attendant public health significance, require further studies.

## References

[CR1] Abe N, Matsubayashi M, Kimata I, Iseki M (2006). Subgenotype analysis of *Cryptosporidium parvum* isolates from humans and animals in Japan using the 60-kDa glycoprotein gene sequences. Parasitol Res.

[CR2] Balicka-Ramisz A, Ramisz S, Snitynskyj V (2012). Prevalence of coccidia infection in goats in Western Pomerania (Poland) and West Ukraine region. Ann Parasitol.

[CR3] Bouix J, Krupinski J, Rzepecki R, Nowosad B, Skrzyzala I, Roborzynski M, Fudalewicz-Niemczyk W, Skalska M, Malczewski A, Gruner L (1998). Genetic resistance to gastrointestinal nematode parasites in Polish long-wool sheep. Int J Parasitol.

[CR4] Brook EJ, Anthony Hart C, French NP, Christley RM (2009). Molecular epidemiology of *Cryptosporidium* subtypes in cattle in England. Vet J.

[CR5] Buddle BM, Herceg M, Ralston MJ, Pulford HD, Millar KR, Elliott DC (1988). A goat mortality study in the southern North Island. N Z Vet J.

[CR6] Cacciò SM, Sannella AR, Mariano V, Valentini S, Berti F, Tosini F, Pozio E (2013). A rare *Cryptosporidium parvum* genotype associated with infection of lambs and zoonotic transmission in Italy. Vet Parasitol.

[CR7] Cieloszyk J, Goñi P, García A, Remacha MA, Sánchez E, Clavel A (2012). Two cases of zoonotic cryptosporidiosis in Spain by the unusual species *Cryptosporidium ubiquitum* and *Cryptosporidium felis*. Enferm Infecc Microbiol Clin.

[CR8] Connelly L, Craig BH, Jones B, Alexander CL (2013). Genetic diversity of *Cryptosporidium* spp. within a remote population of soay sheep on St. Kilda Islands, Scotland. Appl Environ Microbiol.

[CR9] CSO (2010) Working Group of National Agricultural Census of 2010. Livestock and selected elements of animal production methods. Zakład Wydawnictw Statystycznych, Warszawa

[CR10] Delafosse A, Castro-Hermida JA, Baudry C, Ares-Mazás E, Chartier C (2006). Herd-level risk factors for *Cryptosporidium* infection in dairy-goat kids in western France. Prev Vet Med.

[CR11] Díaz P, Quílez J, Prieto A, Navarro E, Pérez-Creo A, Fernández G, Panadero R, López C, Díez-Baños P, Morrondo P (2015). *Cryptosporidium* species and subtype analysis in diarrhoeic pre-weaned lambs and goat kids from north-western Spain. Parasitol Res.

[CR12] Díaz P, Quílez J, Robinson G, Chalmers RM, Díez-Baños P, Morrondo P (2010). Identification of *Cryptosporidium xiaoi* in diarrhoeic goat kids (*Capra hircus*) in Spain. Vet Parasitol.

[CR13] Elwin K, Chalmers RM (2008). Contemporary identification of previously reported novel *Cryptosporidium* isolates reveals *Cryptosporidium bovis* and the cervine genotype in sheep (*Ovis aries*). Parasitol Res.

[CR14] Fiuza VR, Cosendey RI, Frazão-Teixeira E, Santín M, Fayer R, de Oliveira FC (2011). Molecular characterization of *Cryptosporidium* in Brazilian sheep. Vet Parasitol.

[CR15] Geurden T, Thomas P, Casaert S, Vercruysse J, Claerebout E (2008). Prevalence and molecular characterisation of *Cryptosporidium* and *Giardia* in lambs and goat kids in Belgium. Vet Parasitol.

[CR16] Glaberman S, Moore JE, Lowery CJ, Chalmers RM, Sulaiman I, Elwin K, Rooney PJ, Millar BC, Dooley JS, Lal AA, Xiao L (2002). Three drinking-water-associated cryptosporidiosis outbreaks, Northern Ireland. Emerg Infect Dis.

[CR17] Giles M, Chalmers R, Pritchard G, Elwin K, Mueller-Doblies D, Clifton-Hadley F (2009). *Cryptosporidium hominis* in a goat and a sheep in the UK. Vet Rec.

[CR18] Hijjawi N, Ng J, Yang R, Atoum MF, Ryan U (2010). Identification of rare and novel *Cryptosporidium* GP60 subtypes in human isolates from Jordan. Exp Parasitol.

[CR19] Homan W, van Gorkom T, Kan YY, Hepener J (1999). Characterization of *Cryptosporidium parvum* in human and animal feces by single-tube nested polymerase chain reaction and restriction analysis. Parasitol Res.

[CR20] Imre K, Luca C, Costache M, Sala C, Morar A, Morariu S, Ilie MS, Imre M, Dărăbuş G (2013). Zoonotic *Cryptosporidium parvum* in Romanian newborn lambs (*Ovis aries*). Vet Parasitol.

[CR21] Jiang Y, Ren J, Yuan Z, Liu A, Zhao H, Liu H, Chu L, Pan W, Cao J, Lin Y, Shen Y (2014). *Cryptosporidium andersoni* as a novel predominant *Cryptosporidium* species in outpatients with diarrhoea in Jiangsu Province, China. BMC Infect Dis.

[CR22] Kaupke A, Rzeżutka A (2015). Emergence of novel subtypes of *Cryptosporidium parvum* in calves in Poland. Parasitol Res.

[CR23] Kang’ethe EK, Mulinge EK, Skilton RA, Njahira M, Monda JG, Nyongesa C, Mbae CK, Kamwati SK (2012). *Cryptosporidium* species detected in calves and cattle in Dagoretti, Nairobi, Kenya. Trop Anim Health Prod.

[CR24] Karanis P, Plutzer J, Halim NA, Igori K, Nagasawa H, Ongerth J, Liqing M (2007). Molecular characterization of *Cryptosporidium* from animal sources in Qinghai province of China. Parasitol Res.

[CR25] Koinari M, Lymbery AJ, Ryan UM (2014). *Cryptosporidium* species in sheep and goats from Papua New Guinea. Exp Parasitol.

[CR26] Lange H, Johansen OH, Vold L, Robertson LJ, Anthonisen IL, Nygard K (2014). Second outbreak of infection with a rare *Cryptosporidium parvum* genotype in schoolchildren associated with contact with lambs/goat kids at a holiday farm in Norway. Epidemiol Infect.

[CR27] Li P, Cai J, Cai M, Wu W, Li C, Lei M, Xu H, Feng L, Ma J, Feng Y, Xiao L (2016). Distribution of *Cryptosporidium* species in Tibetan sheep and yaks in Qinghai, China. Vet Parasitol.

[CR28] Mahfouz ME, Mira N, Amer S (2014). Prevalence and genotyping of *Cryptosporidium* spp. in farm animals in Egypt. J Vet Med Sci.

[CR29] Majewska AC, Werner A, Sulima P, Luty T (2000). Prevalence of *Cryptosporidium* in sheep and goats bred on five farms in west-central region of Poland. Vet Parasitol.

[CR30] Malczewski A (1970) Gastro-intestinal helminths of ruminants in Poland. I. Helminth fauna of sheep. Acta Parasit Pol 18:245–254

[CR31] Mason RW, Hartley WJ, Tilt L (1981). Intestinal cryptosporidiosis in a kid goat. Aust Vet J.

[CR32] Mi R, Wang X, Huang Y, Zhou P, Liu Y, Chen Y, Chen J, Zhu W, Chen Z (2014). Prevalence and molecular characterization of *Cryptosporidium* in goats across four provincial level areas in China. PLoS One.

[CR33] Misić Z, Katic-Radivojevic S, Kulisic Z (2006). *Cryptosporidium* infection in lambs and goat kids in Serbia. Acta Vet (Beogr).

[CR34] Mueller-Doblies D, Giles M, Elwin K, Smith RP, Clifton-Hadley FA, Chalmers RM (2008). Distribution of *Cryptosporidium* species in sheep in the UK. Vet Parasitol.

[CR35] Ozmen O, Yukari BA, Haligur M, Sahinduran S (2006). Observations and immunohistochemical detection of *Coronavirus*, *Cryptosporidium parvum* and *Giardia intestinalis* in neonatal diarrhoea in lambs and kids. Schweizer Archiv fur Tierheilkunde.

[CR36] Paoletti B, Giangaspero A, Gatti A, Iorio R, Cembalo D, Milillo P, Traversa D (2009). Immunoenzymatic analysis and genetic detection of *Cryptosporidium parvum* in lambs from Italy. Exp Parasitol.

[CR37] Paraud C, Pors I, Rieux A, Brunet S (2014). High excretion of *Cryptosporidium ubiquitum* by peri-parturient goats in one flock in western France. Vet Parasitol.

[CR38] Paz e Silva FM, Lopes RS, Bresciani KD, Amarante AF, Araujo JP (2014). High occurrence of *Cryptosporidium ubiquitum* and *Giardia duodenalis* genotype E in sheep from Brazil. Acta Parasitol.

[CR39] Plutzer J, Karanis P (2009). Genetic polymorphism in *Cryptosporidium* species: an update. Vet Parasitol.

[CR40] Plutzer J, Karanis P (2007). Genotype and subtype analyses of *Cryptosporidium* isolates from cattle in Hungary. Vet Parasitol.

[CR41] Pritchard GC, Marshall JA, Giles M, Mueller-Doblies D, Sayers AR, Marshall RN, Elwin K, Chalmers RM (2008). *Cryptosporidium* species in lambs submitted for diagnostic postmortem examination in England and Wales. Vet Rec.

[CR42] Quílez J, Torres E, Chalmers RM, Hadfield SJ, Del Cacho E, Sánchez-Acedo C (2008). *Cryptosporidium* genotypes and subtypes in lambs and goat kids in Spain. Appl Environ Microbiol.

[CR43] Quílez J, Vergara-Castiblanco C, Monteagudo L, Del Cacho E, Sánchez-Acedo C (2011). Multilocus fragment typing and genetic structure of *Cryptosporidium parvum* isolates from diarrheic preweaned calves in Spain. Appl Environ Microbiol.

[CR44] Rieux A, Paraud C, Pors I, Chartier C (2013). Molecular characterization of *Cryptosporidium* spp. in pre-weaned kids in a dairy goat farm in western France. Vet Parasitol.

[CR45] Robertson LJ (2009). *Giardia* and *Cryptosporidium* infections in sheep and goats: a review of the potential for transmission to humans via environmental contamination. Epidemiol Infect.

[CR46] Robertson LJ, Gjerde BK, Furuseth Hansen E (2010). The zoonotic potential of *Giardia* and *Cryptosporidium* in Norwegian sheep: a longitudinal investigation of 6 flocks of lambs. Vet Parasitol.

[CR47] Romero-Salas D, Alvarado-Esquivel C, Cruz-Romero A, Aguilar-Domínguez M, Ibarra-Priego N, Merino-Charrez JO, Pérez de León AA, Hernández-Tinoco J (2016). Prevalence of *Cryptosporidium* in small ruminants from Veracruz, Mexico. BMC Vet Res.

[CR48] Ryan UM, Bath C, Robertson I, Read C, Elliot A, McInnes L, Traub R, Besier B (2005). Sheep may not be an important zoonotic reservoir for *Cryptosporidium* and *Giardia* parasites. Appl Environ Microbiol.

[CR49] Rzeżutka A, Kaupke A (2013). Occurrence and molecular identification of *Cryptosporidium* species isolated from cattle in Poland. Vet Parasitol.

[CR50] Santín M, Trou JM, Fayer R (2007). Prevalence and molecular characterization of *Cryptosporidium* and *Giardia* species and genotypes in sheep in Maryland. Vet Parasitol.

[CR51] Sharbatkhori M, Nazemalhosseini Mojarad E, Taghipour N, Pagheh AS, Mesgarian F (2015). Prevalence and genetic characterization of *Cryptosporidium* spp. in diarrheic children from Gonbad Kavoos City, Iran. Iran J Parasitol.

[CR52] Shen Y, Yin J, Yuan Z, Lu W, Xu Y, Xiao L, Cao J (2011). The identification of the *Cryptosporidium ubiquitum* in pre-weaned Ovines from Aba Tibetan and Qiang autonomous prefecture in China. Biomed Environ Sci.

[CR53] Silverlås C, Näslund K, Björkman C, Mattsson JG (2010). Molecular characterisation of *Cryptosporidium* isolates from Swedish dairy cattle in relation to age, diarrhoea and region. Vet Parasitol.

[CR54] Soba B, Logar J (2008). Genetic classification of *Cryptosporidium* isolates from humans and calves in Slovenia. Parasitology.

[CR55] Sulaiman IM, Hira PR, Zhou L, Al-Ali FM, Al-Shelahi FA, Shweiki HM, Iqbal J, Khalid N, Xiao L (2005). Unique endemicity of cryptosporidiosis in children in Kuwait. J Clinical Microbiol.

[CR56] Stantic-Pavlinic M, Xiao L, Glaberman S, Lal AA, Orazen T, Rataj-Verglez A, Logar J, Berce I (2003). *Cryptosporidiosis* associated with animal contacts. Wien Klin Wochenschr.

[CR57] Sweeny JP, Ryan UM, Robertson ID, Yang R, Bell K, Jacobson C (2011). Longitudinal investigation of protozoan parasites in meat lamb farms in southern Western Australia. Prev Vet Med.

[CR58] Taylan-Ozkan A, Yasa-Duru S, Usluca S, Lysen C, Ye J, Roellig DM, Feng Y, Xiao L (2016). *Cryptosporidium* species and *Cryptosporidium parvum* subtypes in dairy calves and goat kids reared under traditional farming systems in Turkey. Exp Parasitol.

[CR59] Tzanidakis N, Sotiraki S, Claerebout E, Ehsan A, Voutzourakis N, Kostopoulou D, Stijn C, Vercruysse J, Geurden T (2014). Occurrence and molecular characterization of *Giardia duodenalis* and *Cryptosporidium* spp. in sheep and goats reared under dairy husbandry systems in Greece. Parasite.

[CR60] Vasilkova Z, Krupicer I, Legath J, Kovalkovicova N, Pet’ko B (2004). Coccidiosis of small ruminants in various regions of Slovakia. Acta Parasitol.

[CR61] Wang Y, Feng Y, Cui B, Jian F, Ning C, Wang R, Zhang L, Xiao L (2010). *Cervine* genotype is the major *Cryptosporidium* genotype in sheep in China. Parasitol Res.

[CR62] Wang R, Li G, Cui B, Huang J, Cui Z, Zhang S, Dong H, Yue D, Zhang L, Ning C, Wang M (2014) Prevalence, molecular characterization and zoonotic potential of *Cryptosporidium* spp. in goats in Henan and Chongqing, China. Exp Parasitol 142:11–16 10.1016/j.exppara.2014.04.001 24721256

[CR63] Wielinga PR, de Vries A, van der Goot TH, Mank T, Mars MH, Kortbeek LM, van der Giessen JW (2008). Molecular epidemiology of *Cryptosporidium* in humans and cattle in the Netherlands. Int J Parasitol.

[CR64] Xiao L (2010). Molecular epidemiology of cryptosporidiosis: an update. Exp Parasitol.

[CR65] Xiao L, Escalante L, Yang C, Sulaiman I, Escalante AA, Montali RJ, Fayer R, Lal AA (1999). Phylogenetic analysis of *Cryptosporidium* parasites based on the small-subunit rRNA gene locus. Appl Environ Microbiol.

[CR66] Xiao L, Moore JE, Ukoh U, Gatei W, Lowery CJ, Murphy TM, Dooley JS, Millar BC, Rooney PJ, Rao JR (2006). Prevalence and identity of *Cryptosporidium* spp. in pig slurry. Appl Environ Microbiol.

[CR67] Xiao L, Ryan UM (2004). *Cryptosporidiosis*: an update in molecular epidemiology. Curr Opin Infect Dis.

[CR68] Yang R, Jacobson C, Gardner G, Carmichael I, Campbell AJ, Ryan U (2014). Longitudinal prevalence, oocyst shedding and molecular characterisation of *Cryptosporidium* species in sheep across four states in Australia. Exp Parasitol.

[CR69] Ye J, Xiao L, Wang Y, Wang L, Amer S, Roellig DM, Guo Y, Feng Y (2013). Periparturient transmission of *Cryptosporidium xiaoi* from ewes to lambs. Vet Parasitol.

[CR70] Zintl A, Neville D, Maguire D, Fanning S, Mulcahy G, Smith HV, De Waal T (2007). Prevalence of *Cryptosporidium* species in intensively farmed pigs in Ireland. Parasitol.

